# Immunomodulatory effects of medicinal and edible plants in autoimmune diseases: mechanisms and therapeutic potential

**DOI:** 10.3389/fimmu.2026.1816712

**Published:** 2026-08-03

**Authors:** Zhenzhen Gu, Xi Chen, Shengqi Zhang, Mingzi Sun, Zhongyu Li, Ning Cui

**Affiliations:** 1 Eye Hospital, China Academy of Chinese Medical Sciences, Beijing, China; 2 Graduate School, China Academy of Chinese Medical Sciences, Beijing, China; 3 Graduate School, Beijing University of Chinese Medicine, Beijing, China; 4 Wangjing Hospital, China Academy of Chinese Medical Sciences, Beijing, China; 5 Department of Oncology, Xiyuan Hospital, China Academy of Chinese Medical Sciences, Beijing, China

**Keywords:** autoimmune diseases, clinical translations, immune homeostasis, immunomodulation, inflammatory signaling, medicinal and edible plants

## Abstract

Autoimmune diseases (ADs) and related immune-mediated inflammatory disorders are characterized by loss of immune tolerance, persistent inflammatory activation, immune-cell imbalance, oxidative stress, and tissue-specific injury. Although conventional immunomodulatory therapies remain central to disease management, their long-term use may be limited by adverse effects, variable treatment responses, and high costs. Medicinal and edible plants (MEPs) provide a diverse source of bioactive compounds with potential immunoregulatory activity. This narrative review summarizes the immunological basis of representative autoimmune and immune-mediated inflammatory disorders and critically evaluates the mechanisms, therapeutic potential, and translational limitations of selected MEPs and their major active constituents. Current evidence indicates that MEP-derived interventions commonly regulate NF-κB, MAPK, JAK/STAT, and NOD-like receptor family pyrin domain-containing 3 (NLRP3) inflammasome signaling, while also modulating imbalances in Th17/Treg and Th1/Th2 responses, oxidative stress, and tissue barrier dysfunction. In addition to these shared mechanisms, several disease-relevant regulatory patterns have emerged, including high mobility group box 1 (HMGB1) inhibition in lupus-associated renal injury, regulation of histone lactylation and ferroptosis in rheumatoid arthritis-associated synovial pathology, neutrophil modulation in inflammatory arthritis, and intestinal protection mediated by the microbiota–metabolite–aryl hydrocarbon receptor (AhR) axis in colitis. However, most available evidence is derived from cellular and animal studies, whereas high-quality clinical data remain limited. Poor bioavailability, variable chemical composition, insufficient formulation and dosage standardization, incomplete safety evaluation, and regulatory challenges continue to hinder clinical translation. MEPs therefore represent promising sources of adjunctive immunomodulatory strategies for ADs, but their therapeutic value requires further validation through standardized mechanistic studies and well-designed clinical trials.

## 1 Introduction

Autoimmune diseases (ADs) are pathological conditions caused by immune system dysfunction, characterized by the immune system mistakenly attacking self-tissues (1, 2). ADs are highly heterogeneous, with at least 80 types identified to date (3). Recent decades have seen a notable rise in the prevalence of ADs (4). It has been estimated that the global incidence and prevalence of ADs are increasing annually at rates of 19.1% and 12.5%, respectively (5). Currently, approximately 11-15% of the global population is affected by ADs, including conditions such as psoriasis, rheumatoid arthritis (RA), systemic lupus erythematosus (SLE), multiple sclerosis (MS), and ulcerative colitis (UC). Although immunosuppressive agents are commonly used clinical therapies (6), their long-term use may cause severe adverse effects, such as an increased risk of infection and metabolic disorders, while some agents are also associated with high treatment costs (7). Consequently, an increasing number of patients are turning to natural products to alleviate disease-related symptoms (8). More than 36% of adults in the United States have reported using complementary and alternative medicine (9), and herbal medicines have attracted increasing attention as complementary approaches for rheumatic diseases (10). In parallel, bioactive components derived from medicinal and edible plants (MEPs), particularly polysaccharides, have been increasingly investigated for their potential roles in the management of chronic diseases (11). These findings support further exploration of MEP-derived interventions for immune-mediated disorders. MEPs with both nutritional and pharmacological activities hold significant potential for application in pharmaceuticals, functional foods, and health supplements. Their diverse bioactive components can regulate immune homeostasis through multi-target mechanisms, thereby providing novel perspectives for the prevention and treatment of ADs (12). Some MEP-derived interventions may have therapeutic potential in ADs by modulating immune homeostasis and suppressing pathological immune activation rather than broadly enhancing immune responses (13). As of 2024, the National Health Commission of China has officially listed 106 MEPs. This review systematically summarizes the mechanisms underlying immune dysregulation in ADs and critically discusses the immunomodulatory mechanisms, therapeutic potential, and translational challenges of MEPs in the prevention and treatment of ADs.

## 2 Immunological dysregulation mechanisms in ADs

### 2.1 Imbalance of immune homeostasis

The immune system protects the host against pathogens and harmful stimuli while supporting the clearance of damaged cells and tissue repair. It must maintain a balance between immune defense and immune tolerance. Impaired immune function may increase susceptibility to severe infections or tumor development, whereas excessive immune activation may trigger allergies or ADs (14). Under normal physiological conditions, the immune system distinguishes self-antigens from foreign antigens through self-tolerance mechanisms, thereby preventing attacks on the body’s own tissues. However, when these mechanisms become dysfunctional, immune responses against self-antigens may arise and contribute to the development of ADs (15). Pathogenic autoimmunity arises when specific self-molecules trigger significant inflammatory responses mediated by self-reactive T cells, B cells, or autoantibodies, thereby recruiting both innate and adaptive immune cells. These inflammatory responses may be confined to a single organ, such as autoimmune thyroiditis, or may involve multiple organ systems throughout the body (16). The development of ADs can be broadly conceptualized as involving three stages. First, during the initiation stage, genetic and environmental factors lead to the loss of immune tolerance and subsequent activation of self-reactive T and B lymphocytes. Second, the transitional stage is characterized by immune complex deposition and immune cell recruitment. Finally, during the effector stage, various immune cells become activated, resulting in tissue damage and chronic inflammation (16).

### 2.2 Molecular pathological characteristics of ADs

Immune cell subsets, including T helper 1 (Th1), Th2, Th17, regulatory T (Treg), regulatory B (Breg), and natural killer (NK) cells, participate in the balance between immune tolerance and inflammatory responses (17, 18). Treg and Breg cells contribute to the maintenance of immune tolerance, partly through the production of inhibitory cytokines such as interleukin-10 (IL-10) and transforming growth factor-β (TGF-β). In contrast, excessive Th1- and Th17-associated responses may promote autoimmune inflammation through the production of interferon-γ (IFN-γ) and IL-17, respectively. Th2 responses are characterized by the secretion of IL-4, IL-5, and IL-13 and may contribute to disease-specific humoral immune abnormalities. In addition, dysregulated B-cell responses can promote autoantibody production and immune-mediated tissue injury (19, 20). Disruption of the balance between effector and regulatory immune cells may therefore facilitate the loss of immune tolerance and the development of ADs (19).

Furthermore, accumulating evidence has demonstrated a close association between innate immune responses and the development of ADs. Under physiological conditions, phagocytes efficiently clear apoptotic cells and cellular debris. In ADs, impaired clearance of apoptotic cells may lead to the accumulation of self-antigens and the initiation of autoimmune responses (21). In addition, dysregulation of macrophage polarization (M1/M2) and Toll-like receptor (TLR) signaling exacerbates inflammatory responses (22). Activation of inflammasome pathways, particularly the NOD-like receptor family pyrin domain-containing 3 (NLRP3) inflammasome, may amplify immune-mediated inflammation through the release of IL-1β and IL-18, and has been implicated in disease-associated tissue injury in conditions such as UC and lupus nephritis (23, 24).

Cytokines are key mediators involved in the initiation and regulation of immune responses. Classical macrophage activation is initiated in response to tissue injury or infection, triggered by microbial products or pro-inflammatory cytokines, including bacterial lipopolysaccharide (LPS), IFN-γ, and tumor necrosis factor-α (TNF-α). This activation results in the triggering of inflammatory signaling pathways, particularly nuclear factor kappa B (NF-κB), mitogen-activated protein kinase (MAPK), and Janus kinase/signal transducer and activator of transcription (JAK/STAT) pathways (22). Aberrant activation of these pathways promotes the release of pro-inflammatory mediators, including TNF-α, IL-1β, and IL-6 (25). TNF-α has been identified as a pivotal regulatory factor in the immune system. It plays a crucial role in the maturation and recruitment of dendritic cells via the TNFR1 pathway, while also inducing the release of chemokines and cytokines through Toll-like receptor and NF-κB signaling pathways. These processes promote the recruitment and activation of neutrophils, macrophages, and lymphocytes, highlighting the pivotal role of TNF-α in orchestrating inflammatory responses in autoimmune conditions (26).

### 2.3 Pathogenesis of ADs

ADs constitute a broad spectrum of at least 80 distinct pathological entities that share common molecular mechanisms, namely pathological immune responses against the host tissues mediated by immune system dysfunction (3). This section summarizes the pathogenic mechanisms of six autoimmune or immune-mediated inflammatory diseases considered in this review.

SLE is a multiorgan, multisystem autoimmune disease whose pathogenesis involves elevated levels of type I interferons, the production of autoantibodies against nuclear antigens (such as double-stranded DNA and nucleic acid-binding proteins), immune complex deposition, and systemic inflammation affecting target organs including the skin, joints, and kidneys (27, 28). The pathogenesis of RA is highly complex. Within the innate immune system, neutrophil extracellular traps promote osteoclast differentiation and exacerbate bone destruction through non-classical pathways, while mast cells contribute to synovial inflammation and tissue remodeling (29). In the adaptive immune response, dysregulation of Th17/Treg balance and abnormal expression of the LAT1 transporter promote inflammatory progression. In addition, fatty acid metabolic reprogramming in CD8+ T cells significantly enhances their pro-inflammatory properties (29). The core pathogenesis of MS involves the abnormal activation of autoreactive T cells in the peripheral immune system, followed by their migration into the central nervous system (CNS), where they trigger neuroinflammation and demyelination (30). This process begins when dendritic cells and macrophages present self-antigens to CD4+ T cells, leading to their activation. Activated T cells subsequently secrete pro-inflammatory cytokines and matrix metalloproteinases (MMPs), thereby disrupting the blood-brain barrier (BBB). BBB disruption facilitates the infiltration of immune cells into the CNS, where inflammatory responses involving B cells, macrophages, cytotoxic CD8+ T cells, and activated microglia further promote demyelination and neuroaxonal injury (31, 32).

Psoriasis is a chronic immune-mediated inflammatory skin disease characterized by abnormal keratinocyte proliferation and sustained activation of the IL-23/IL-17 axis. Additional activation of NF-κB and JAK/STAT signaling further promotes cytokine production and amplifies cutaneous inflammation (33). The central pathogenic mechanism of autoimmune myocarditis (AM) involves autoreactive T cells targeting cardiac antigens, such as α-myosin heavy chain, that escape thymic negative selection and infiltrate the myocardium, thereby triggering local inflammatory responses. Simultaneously, infiltration of monocytes and macrophages creates a pathological immune microenvironment, collectively contributing to myocardial injury and cardiac dysfunction (34). The pathogenesis of UC is characterized by intricate interplay among genetic susceptibility, immune dysregulation, and gut microbiota imbalance, leading to disruption of the intestinal mucosal barrier and exacerbation of intestinal inflammation (35).

Although these autoimmune and immune-mediated inflammatory diseases affect distinct organs and tissues, they share several immunopathological mechanisms, including aberrant activation of NF-κB, MAPK, and JAK/STAT signaling pathways, dysregulated macrophage polarization, NLRP3 inflammasome activation, and Th17/Treg imbalance. Increasing evidence indicates that natural products derived from medicinal and edible materials may exert therapeutic effects by modulating these conserved inflammatory and immune-regulatory pathways. Understanding the mechanistic overlap between such bioactive constituents and immune dysregulation may therefore support the development of novel multitarget therapeutic strategies.

## 3 Representative medicinal and edible plants and their mechanisms of action

**Figure 1:**
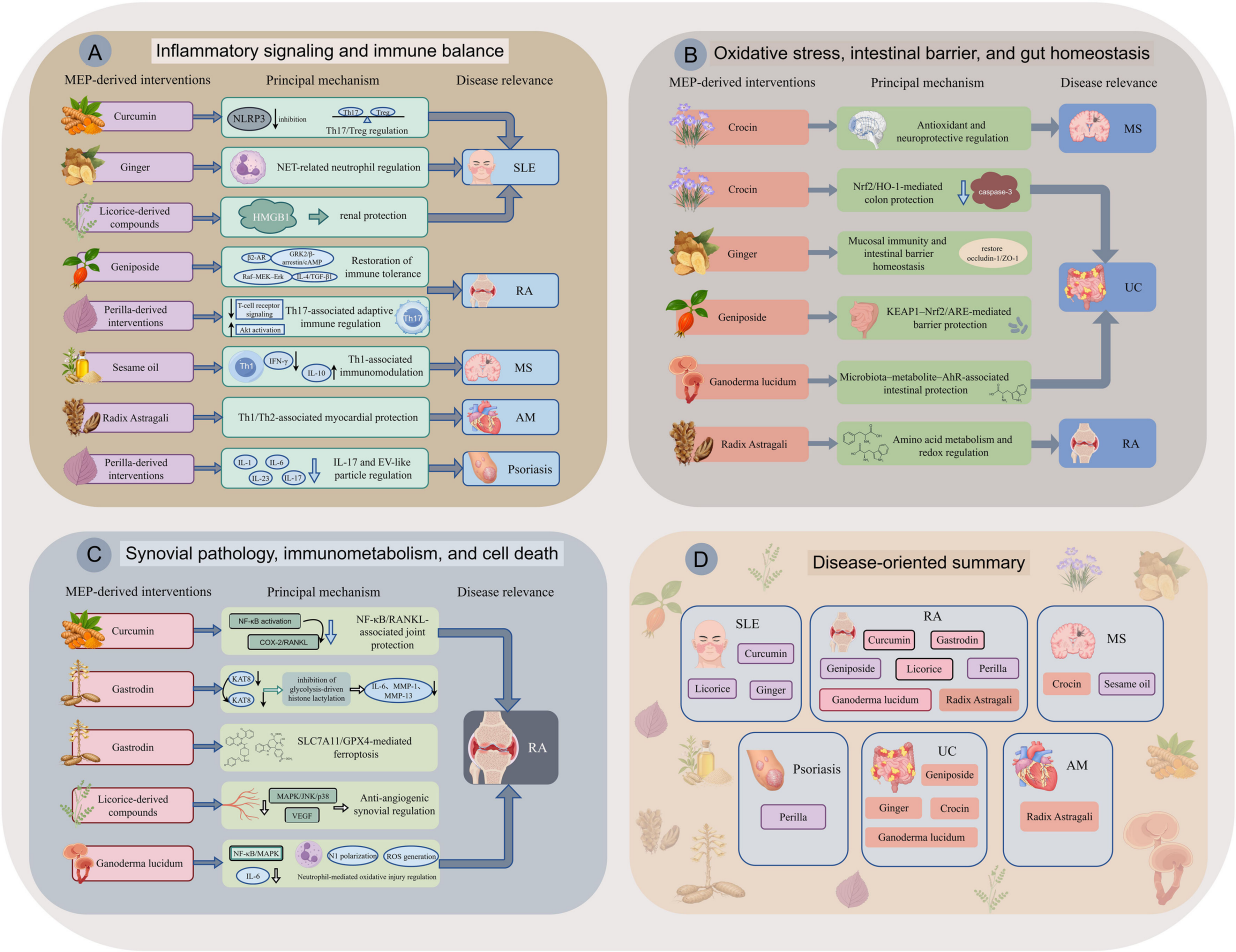
Mechanistic landscape and disease relevance of representative medicinal and edible plant (MEP)-derived interventions in autoimmune diseases. AhR, aryl hydrocarbon receptor; AM, autoimmune myocarditis; ARE, antioxidant response element; GPX4, glutathione peroxidase 4; HMGB1, high-mobility group box 1; HO-1, heme oxygenase-1; KEAP1, Kelch-like ECH-associated protein 1; MEP, medicinal and edible plant; MS, multiple sclerosis; NET, neutrophil extracellular trap; NF-κB, nuclear factor kappa B; NLRP3, NOD-like receptor family pyrin domain-containing 3; Nrf2, nuclear factor erythroid 2-related factor 2; RA, rheumatoid arthritis; RANKL, receptor activator of nuclear factor-κB ligand; SLE, systemic lupus erythematosus; UC, ulcerative colitis.

The animal studies of MEP-derived interventions discussed in this review are detailed in **Table 1**, while their principal mechanistic links to autoimmune diseases are schematically summarized in **Figure 1**.

**Table 1:** Animal model data on the treatment of autoimmune diseases using medicinal plants mentioned in the article.

ADs	Components	Plant source	Experimental model	Intervention dose	Comparator/control	Duration	Core mechanism	References
Systemic lupus erythematosus	curcumin	*Curcuma longa* L.	Human monocytes	20 μM	lipopolysaccharide	7 days	Inhibition of NLRP3 inflammasome activation	(23)
	curcumin	*Curcuma longa* L.	Female NZBWF1 mice	500 mg/kg/day	NZW/LacJ mice	14 days	Modulation of autoimmune activity and attenuation of renal injury	(46)
	6-gingerol	*Zingiber officinale* Roscoe	C57BL/6 mice	10 mg/kg, 20 mg/kg	None	not mentioned	Phosphodiesterase inhibition-mediated suppression of neutrophil hyperactivity	(65)
	glycyrrhizin	Glycyrrhiza glabra	female BALB/c mice	10 mg/kg	Blank Control Group	12 weeks	Blockade of extracellular HMGB1-mediated ALD-DNA endosomal accumulation and macrophage activation	(111)
Rheumatoid Arthritis	curcuminoid-containing turmeric extract	*Curcuma longa* L.	female Lewis rats	23 and/or 46 mg curcuminoids/kg/day	saline	28 days	Inhibition of articular NF-κB activation and NF-κB-regulated chemokine/COX-2/RANKL expression	(48)
	crocin	*Crocus sativus* L.	Wistar rats	10, 20 or 40 mg/kg	saline	36 days	Suppression of MMPs and pro-inflammatory cytokine expression	(55)
	crocin	*Crocus sativus* L.	Wistar SD rats	6.25, 12.5 or 25 mg/kg	methotrexate	28 days	Suppression of iNOS and TNF-α/IL-1β/IL-6 production	(56)
	Zingerone	*Zingiber officinale* Roscoe	Wistar rats	25 mg/kg	Blank Control Group	3 weeks	Suppression of NF-κB-associated inflammatory cytokine responses and restoration of antioxidant defenses	(67)
	Geniposide	*Gardenia jasminoides* Ellis	Female Wistar rats	33, 66 and 132 mg/kg	Sinomenine(90 mg/kg)	15 days	Raf/MEK/Erk1/2 suppression with IL-17/IL-4/TGF-β1 cytokine modulation	(84)
	Geniposide	*Gardenia jasminoides* Ellis	Sprague-Dawley rats	30, 60 and 120 mg	Equal volume of water	7 days	β2-AR/GRK2/β-arrestin/cAMP-associated enhancement of immune tolerance in MLN lymphocytes	(85)
	gastrodin	*Gastrodia elata*	SD male rats	20 mg/kg	dexamethasone (DEX) (5 mg/kg)	50 days	KAT8/H3K9la-associated immunometabolic regulation	(96)
	Sesame Oil	*Sesamum indicum* L.	Male Wistar rat	1.0 mL/rat/da	Dietary oils without minor components	30 days	Minor component-dependent suppression of oxidative stress and inflammatory mediators	(106)
	*Ganoderma lucidum* polysaccharide peptide	Leyss.ex Fr.	Male Wistar rats	100 or 200 mg/kg	methotrexate	35 days	NF-κB/MAPK pathway inhibition via reduced ERK1/2 phosphorylation	(117)
	*Ganoderma lucidum* spore oil	Leyss.ex Fr.	DBA/1 J mice	6 mg/Kg	PEG-400	twice a week for seven weeks	Suppression of IL-6-associated joint inflammation and cartilage degeneration	(118)
	*Ganoderma lucidum* spore powder	Leyss.ex Fr.	Male C57BL/6 mice	50 mg/mloral gavage	double-distilled water	Starting 3 weeks after immunization until the end of the experiment	TNF-α-associated inhibition of neutrophil N1 polarization and ROS production	(119)
	*Radix Astragali* extraction	*Radix Astragali*	male Sprague-Dawley rats	2.1 g crude AstragaliRadix per kilogram per day	Blank Control Group	21 days	Tryptophan/phenylalanine metabolism regulation and redox homeostasis restoration	(127)
Multiple sclerosis	curcumin	*Curcuma longa* L.	Female C57BL/6 mice	10 mg/kg	rapamycin	15 days	Akt/mTOR-associated autophagy regulation	(50)
	curcumin	*Curcuma longa* L.	Female C57BL/6 mice	100 μg	0 μg curcumin	14 days	Th1/Th17 suppression with IL-10/Treg response enhancement	(51)
	ethanol extract of Saffron	*Crocus sativus* L.	Male C57BL/6 mice	500 mg/kg	100 μL double distilled water	21 days	Enhancement of antioxidant capacity with suppression of spinal cord leukocyte infiltration	(57)
	Ginger Extract	*Zingiber officinale* Roscoe	female C57BL6 mice	300 mg/kg	Blank Control Group	21 days	IL-17/IFN-γ suppression with Treg enhancement and NO reduction	(71)
	sesame oil	*Sesamum indicum* L.	Male C57BL/6 mice	4 mL/kg/day	phosphate‐buffered saline	25 days	Th1-associated immunomodulation through IFN-γ suppression and IL-10 enhancement	(108)
	Ethanol extract of Glycyrrhizae Radix modulates	*Glycyrrhiza uralensis*	Female C57BL/6 mice	100, 250, 500 mg/kg	FTY720(3 mg/kg/day)	9 days	Suppression of IFN-γ-related Th1/Tc1 responses and microglial inflammatory activation	(113)
Psoriasis	Ginger Extract	*Zingiber officinale* Roscoe	Male mice	100 and 200 mg/kg	1 ml of distilledwater and betamethasone topical ointment	21 days	Metformin and ginger co-loaded liposomes	(73)
	Essential oil extract	*Perilla frutescens* (L.) Britt.	BALB/c mice	62.5 mg/d	IMQ cream and tacrolimus ointment	7 d	Suppression of neutrophil activation and IL-23/IL-17/NF-κB-associated cutaneous inflammation	(102)
Autoimmune myocarditis	*Radix Astragali* injection	*Radix Astragali*	Lewis rats	0.2 ml/100g	potassium phosphate buffer	21 days	Th1/Th2 cytokine balance restoration-mediated cardioprotection	(126)
Ulcerative colitis	Crocin	*Crocus sativus* L.	male Sprague Dawley rats	20 mg/kg	Felodipine(3 mg/kg, orally)	8 days	Nrf2/HO-1-mediated antioxidant colonic protection	(59)
	Crocin	*Crocus sativus* L.	C57BL/6 male mice	50 ppm, 200 ppm	Blank Control Group	21 days	Anti-inflammatory attenuation of colonic injury	(60)
	Edible ginger-derived nanoparticles	*Zingiber officinale* Roscoe	Female C57BL/6 or FVB/NJ mice	0.3 mg/mouse	Blank Control Group	28 days	Intestinal epithelial repair and cytokine regulation by ginger-derived nanoparticles	(74)
	ginger	*Zingiber officinale* Roscoe	Male BALB/c mice	500 mg/kg	normal saline	not mentioned	Gut microbiota diversity and functional homeostasis restoration	(75)
	ginger polysaccharides	*Zingiber officinale* Roscoe	male C57BL/6 mice	100 mg/Kg, 100 mg/kg	normal drinking water	10 days	Tight-junction restoration and gut microbiota modulation	(76)
	*Gardenia jasminoides* Ellis fruit extracts	*Gardenia jasminoides* Ellis	male Sprague-Dawley (SD) rats	50 mg/kg, 100 mg/kg	Sulfasalazine(500 mg/kg)	7 days	Anti-inflammatory and antioxidant regulation by gardenia fruit extract	(86)
	Geniposide	*Gardenia jasminoides* Ellis	Male C57BL/6 mice	20 mg/kg/d, 60 mg/kg/d	distilled water	14 days	KEAP1-Nrf2/ARE-mediated barrier protection	(87)
	Geniposide	*Gardenia jasminoides* Ellis	Male Sprague-Dawley (SD) rats	25, 50 mg/kg	sulfasalazine	14 days	AMPK/MLCK-associated tight-junction restoration	(88)
	polysaccharide GP	*Gastrodia elata*	Male C57BL/6 J mice	200 mg/kg/d	5-aminosalicylic acid	8 days	NLRP3/ASC suppression with barrier and microbiota restoration	(98)
	β-D-glucans	*Ganoderma lucidum*	Male C57BL/6 mice	200 mg/kg	saline	7 days	Intestinal immune and microbiota regulation by β-D-glucans	(121)
	Ganoderic acid A	*Ganoderma lucidum*	male C57BL/6 mice,	20 mg/kg	distilled water	28 days	Microbiota-tryptophan metabolism-AhR axis regulation	(122)
	Ganoderic acid A	*Ganoderma lucidum*	Male C57BL/6J mice	16.5 mg/kg, 50 mg/kg, 150 mg/kg	Sulfasalazine(150 mg/kg)	7 days	Gut microbiota-mediated intestinal barrier restoration	(123)

### 3.1 *Curcuma longa* L.

Turmeric, derived from the desiccated rhizome of *Curcuma longa* L., a tropical plant native to India and Southeast Asia, has been utilized for millennia as a spice, coloring agent, and therapeutic substance (36). Curcumin, a lipophilic diarylheptanoid polyphenol, is generally regarded as the principal bioactive constituent of turmeric and accounts for many of its anti-inflammatory and immunomodulatory effects (37, 38). Among MEP-derived compounds, curcumin has received particular attention in ADs because it targets several disease-relevant immune pathways rather than a single inflammatory mediator (2, 38) At the molecular level, curcumin suppresses key inflammatory pathways involved in autoimmune pathology, including NF-κB, MAPK, JAK/STAT, STAT3, β-catenin, and Notch-1 signaling, thereby reducing the production of IL-1β, TNF-α, IL-6, and IL-17, as well as inflammatory enzymes such as cyclooxygenase-2 (COX-2) (39–42). Additionally, curcumin regulates macrophage polarization through the phosphoinositide 3-kinase/protein kinase B (PI3K/Akt) pathway, limits pro-inflammatory M1 macrophage activation, and helps restore the Th17/Treg balance (43–45). These effects suggest that curcumin does not simply act as a general anti-inflammatory agent, but may contribute to the re-establishment of immune tolerance in autoimmune inflammation (45).

The therapeutic relevance of curcumin varies across different ADs according to disease-specific pathogenic pathways. In SLE models, curcumin reduced proteinuria, renal inflammation, splenomegaly, and NLRP3 inflammasome activation, indicating potential relevance to lupus nephritis and inflammasome-driven renal injury (23, 46). Its ability to regulate Th17/Treg balance in CD4+ T-cell cultures derived from patients with SLE further supports its potential relevance to adaptive immune dysregulation in this disease (47). In animal models of RA, curcumin extract lacking essential oils significantly inhibited joint inflammation and periarticular tissue destruction, mainly through suppression of local NF-κB activation and downregulation of NF-κB-regulated mediators, including chemokines, COX-2, and receptor activator of nuclear factor kappa-B ligand (RANKL) (48). These preclinical findings indicate that curcumin may be especially relevant to ADs characterized by excessive NF-κB activation, osteoimmune damage, and Th17/Treg imbalance. Although clinical evidence in autoimmune arthritis remains limited, studies in knee osteoarthritis have shown that curcumin may provide symptom relief comparable to ibuprofen with fewer gastrointestinal adverse effects, supporting its relatively favorable safety profile as a plant-derived intervention (49).

Curcumin has also shown neuroprotective and immunoregulatory effects in MS-related preclinical models. Experimental autoimmune encephalomyelitis (EAE) is a widely used animal model for MS. In EAE mice, curcumin regulated Akt/mechanistic target of rapamycin (mTOR) signaling and restored autophagic balance in the central nervous system and peripheral tissues (50). It also reduced IFN-γ, IL-17, IL-12, and IL-23 levels in the CNS and lymphoid organs, while increasing IL-10, and Treg cells, thereby attenuating EAE progression (51). In psoriasis, curcumin may alleviate inflammatory skin lesions by inhibiting phosphorylase kinase activity, regulating keratinocyte proliferation, reducing pro-inflammatory cytokine release, and suppressing TNF-α-mediated apoptosis (52).

Thus, although curcumin shows broad activity across multiple ADs, its dominant mechanisms may vary by disease context: Th17/Treg and NLRP3 regulation appear more relevant in SLE and MS, whereas NF-κB/RANKL and keratinocyte-associated inflammatory pathways may be more important in RA and psoriasis. However, its clinical translation remains limited by poor aqueous solubility, low oral bioavailability, rapid metabolism, and heterogeneity among curcumin formulations. Therefore, the therapeutic potential of curcumin in ADs should be interpreted as formulation-dependent and requires further validation through optimized delivery systems and well-designed clinical trials.

### 3.2 *Crocus sativus* L.

*Crocus sativus* L. is an MEP whose stigmas yield saffron and contain various bioactive constituents, including anthocyanins, flavonoids, terpenes, and carotenoids (21). Among these compounds, crocin is considered one of the major pharmacologically active constituents and exhibits prominent antioxidant and immunomodulatory activities. Recent reviews have highlighted crocin as a saffron-derived phytochemical with therapeutic potential in autoimmune and inflammatory diseases (53). Its distinctive mechanism appears to involve the coordinated regulation of oxidative stress and inflammatory signaling, particularly through the NF-κB and nuclear factor erythroid 2-related factor 2 (Nrf2)/heme oxygenase-1 (HO-1) pathways. Furthermore, crocin inhibits enzymes involved in bone and cartilage degradation, suggesting potential protective effects against osteoimmune-mediated tissue destruction in arthritis (54).

In RA models, crocin significantly reduced paw swelling and arthritis severity through suppression of inducible nitric oxide synthase (iNOS) expression and pro-inflammatory cytokine production (55, 56). In MS-related studies, *Crocus sativus* extracts prevented EAE progression in C57BL/6 mice by reducing oxidative stress and inhibiting leukocyte infiltration into the CNS (57). Unlike curcumin, which predominantly modulates Th17/Treg balance and NF-κB signaling, the effects of saffron-derived interventions in MS-related models appear to rely more heavily on antioxidant defense and neuroprotective mechanisms. Consistently, regulation of NF-κB and Nrf2/HO-1 signaling has been proposed as an important mechanism underlying the anti-inflammatory and immunoregulatory properties of saffron-derived compounds (58). In UC models, crocin demonstrated marked anti-ulcerative and colon-protective effects through antioxidant, anti-inflammatory, and anti-apoptotic activities. These effects were associated with activation of the Nrf2/HO-1 signaling pathway and suppression of caspase-3 activity (59). Another study showed that crocin reduced the colitis disease activity index in mice, including weight loss, diarrhea, rectal bleeding, and colon shortening (60).

Taken together, crocin may be particularly relevant for ADs characterized by oxidative stress, neuroinflammation, or epithelial barrier injury. However, most evidence remains preclinical, and its clinical application is still limited by insufficient disease-specific trials and unresolved pharmacokinetic issues. Further studies are needed to clarify its bioavailability, optimal dosage, and therapeutic efficacy in different AD contexts.

### 3.3 Zingiber officinale Roscoe

Ginger (*Zingiber officinale Roscoe*), a member of the Zingiberaceae family, is a widely utilized medicinal plant in China (61). Ginger-derived compounds, particularly gingerols, shogaols, and sesquiterpenes, modulate autoimmune inflammation by affecting cytokine production, oxidative stress, inflammasome activation, and intestinal barrier function (62, 63). These constituents have been shown to inhibit the production of TNF-α, IL-1β, IL-2, IL-4, IL-6, and IL-17, while reducing the expression of MMP-1, MMP-3, and MMP-13 (64).

In experimental models of lupus and antiphospholipid syndrome, 6-gingerol attenuated neutrophil extracellular trap release by partially inhibiting phosphodiesterase activity, while reducing autoantibody formation and large-vein thrombosis in mice, suggesting its potential to alleviate neutrophil-driven autoimmune inflammation (65). In patients with active RA, ginger supplementation increased FoxP3 expression and reduced RORγt and T-bet expression, suggesting a potential regulatory effect on T-cell balance; however, these findings require confirmation in larger clinical studies with disease activity outcomes (66). Another study demonstrated that zingerone, an active constituent of ginger, also reduced NF-κB, TGF-β, TNF-α, IL-1β, IL-6, and high-sensitivity C-reactive protein levels while increasing IL-10 expression, thereby attenuating inflammation and oxidative stress in RA models (67). Nevertheless, evidence in RA and SLE remains largely preclinical or is derived from small-scale investigations (68).

In MS-related models, ginger and its bioactive components have been reported to modulate cytokine and chemokine production, T-cell subsets, B-cell and macrophage responses, and TLR signaling, while suppressing inflammasome activation and oxidative stress (69–71). Inhibition of the NLRP3 inflammasome may therefore contribute to the neuroprotective effects of ginger-derived compounds in autoimmune neuroinflammation (72). Psoriasis is a chronic autoimmune inflammatory skin disease. In a psoriasis model, liposomes co-loaded with metformin and ginger alleviated psoriasis-like lesions and reduced IL-22 and TNF-α levels (73).

A particularly relevant aspect of ginger is its activity in intestinal inflammatory disease. In a colitis model, oral administration of edible ginger-derived nanoparticles (GDNPs 2) enhanced intestinal epithelial cell survival and proliferation, reduced levels of pro-inflammatory cytokines, and increased anti-inflammatory cytokine production (74). *In vitro* studies further showed that ginger and its extracts protected RSL3-challenged Caco-2 cells by enhancing antioxidant capacity, suppressing pro-inflammatory cytokine production, restoring occludin-1 and ZO-1 expression, and inhibiting ferroptosis. In experimental colitis models, ginger-derived interventions have also been associated with modulation of the intestinal microbiota composition (75–78). Recent reviews have further emphasized that maintenance of epithelial barrier integrity and gut immune homeostasis may be central to the activity of ginger in UC (63, 79).

Overall, ginger is distinguished by its effects on mucosal immunity and intestinal barrier homeostasis, together with additional regulation of inflammatory signaling and innate immune activation. Its therapeutic potential appears particularly relevant to UC and other ADs involving barrier dysfunction. However, clinical evidence remains limited, and further studies are needed to define its active constituents, optimal formulations, and disease-specific efficacy.

### 3.4 *Gardenia jasminoides* Ellis

*Gardenia jasminoides* Ellis is an evergreen shrub belonging to the Rubiaceae family, and its dried fruits are widely used in traditional herbal medicine for the treatment of various diseases (80). Geniposide (GE), one of the principal iridoid glycosides isolated from *Gardenia jasminoides*, has attracted increasing attention because of its anti-inflammatory, antioxidant, and immunomodulatory activities (81). Pharmacological studies indicate that GE modulates NF-κB, Nrf2, MAPK, and AMP-activated protein kinase (AMPK)-related signaling pathways, suggesting its potential relevance to immune-mediated inflammatory diseases (82). In RA-related models, GE suppresses TNF-α-stimulated synovial cell proliferation by upregulating miRNA-124a expression and inhibiting the integrin β1 (Itgβ1)-mediated Ras/Erk1/2 signaling pathway, suggesting a role in limiting pathological synovial proliferation in RA (83). In collagen-induced arthritis (CIA) rats, GE also alleviates pathological damage in mesenteric lymph nodes by inhibiting IL-6 and IL-17 expression, promoting IL-4 and TGF-β1 secretion, and downregulating the Raf-MEK-Erk signaling pathway (84). Furthermore, in adjuvant-induced arthritis (AIA) rats, GE enhanced immune tolerance in mesenteric lymph node (MLN) lymphocytes by upregulating β2-adrenergic receptor (β2-AR) expression, inhibiting G protein-coupled receptor kinase 2 (GRK2)/β-arrestin-mediated desensitization, and regulating the cyclic adenosine monophosphate (cAMP) signaling pathway. These effects were accompanied by reduced IL-2 levels and increased IL-4 and TGF-β1 levels, leading to alleviation of arthritic symptoms (85). Thus, restoration of immune tolerance and suppression of Th17-associated inflammation may represent characteristic mechanisms of GE in RA.

In a 2,4,6-trinitrobenzene sulfonic acid (TNBS)-induced colitis rat model, gardenia fruit extract (GFE) exhibited significant anti-inflammatory and antioxidant activities, as evidenced by reduced levels of IL-1β, TNF-α, IL-6, malondialdehyde (MDA), and nitric oxide (NO), together with increased superoxide dismutase (SOD) activity (86). As GFE is a multicomponent preparation, these protective effects may reflect interactions among its active constituents. Mechanistic studies focusing on GE have shown that it alleviates dextran sulfate sodium (DSS)-induced colitis by interacting with kelch-like ECH-associated protein 1 (KEAP1), stabilizing Nrf2, and activating the Nrf2/antioxidant response element (ARE) antioxidant pathway while suppressing NF-κB signaling, thereby restoring redox homeostasis, reducing lipid peroxidation, inhibiting inflammatory cytokine production, and improving intestinal barrier integrity (87). Notably, these protective effects were completely reversed by the Nrf2 inhibitor ML385, further supporting a central role for the KEAP1-Nrf2/ARE axis in GE-mediated colonic protection (87). Another study revealed that GE repairs TNBS-induced intestinal epithelial barrier damage by activating the AMPK signaling pathway, downregulating the expression of NF-κB, COX-2, iNOS, and myosin light chain kinase (MLCK), while upregulating tight junction protein expression (88). These findings indicate that restoration of redox homeostasis and preservation of epithelial tight junctions are central to the protective effects of GE in colitis (24, 89).

Taken together, *Gardenia jasminoides* and geniposide may act through two disease-relevant mechanisms: restoration of immune tolerance in RA and protection of intestinal barrier homeostasis through KEAP1-Nrf2/ARE-associated antioxidant responses in UC. However, the available evidence remains predominantly preclinical, and further studies are required to determine effective dosing, safety, and clinical applicability.

### 3.5 *Gastrodia elata* Bl.

*Gastrodia elata* Bl. (GEB) is a widely used medicinal plant in traditional Chinese medicine, and its bioactive constituents have demonstrated considerable therapeutic potential in inflammatory diseases (90). Gastrodin (GAS), the major phenolic glycoside isolated from GEB, has attracted increasing attention because of its anti-inflammatory, antioxidant, and immunomodulatory properties (91, 92). For gastrodin, current evidence highlights immunometabolic reprogramming, histone lactylation, and ferroptosis-associated synovial pathology as particularly relevant mechanisms in RA (91–94).

An *in vitro* study demonstrated that GEB effectively reduced the expression of inflammatory cytokines (IL-6 and IL-8) as well as MMP-3 and MMP-13 in TNF-α-induced RA fibroblast-like synoviocytes (RA-FLS) through inhibition of the NF-κB signaling pathway, suggesting its potential therapeutic value in RA (95). Further evidence showed that GAS directly targets and destabilizes KAT8, thereby reducing histone H3K9 lactylation (H3K9la) (96). *In vitro*, GAS suppressed LPS-induced expression of IL-6, MMP-1, and MMP-13 in FLS and THP-1 macrophages by reducing glycolysis and lactate production; *in vivo*, it alleviated joint swelling and synovial hyperplasia in a Sprague–Dawley rat AIA model, accompanied by decreased H3K9la and IL-6 expression (96). These findings suggest that inhibition of glycolysis-driven histone lactylation may be an important mechanism by which gastrodin ameliorates inflammatory synovial pathology in RA (97).

Ferroptosis regulation represents another notable mechanism underlying the anti-arthritic effects of gastrodin. GAS facilitated LPS-induced ferroptosis in RA-FLS by downregulating solute carrier family 7 member 11 (SLC7A11) and inhibiting glutathione peroxidase 4 (GPX4), thereby suppressing abnormal synoviocyte proliferation and ameliorating arthritis in a rat adjuvant arthritis model (92). Thus, gastrodin may limit pathological synovial hyperplasia in RA by targeting both lactylation-associated immunometabolic abnormalities and ferroptosis resistance. In UC, the available evidence mainly concerns GEB-derived polysaccharides rather than gastrodin itself. A glucan polysaccharide isolated from GEB ameliorated DSS-induced colitis in mice by reducing pro-inflammatory mediators, including TNF-α, IL-1β, NLRP3, and apoptosis-associated speck-like protein containing a CARD (ASC), increasing IL-10 expression, enhancing intestinal barrier protein expression, and modulating the gut microbiota (98). These findings indicate that the therapeutic effects of GEB in UC may involve simultaneous regulation of inflammasome activation, intestinal barrier integrity, and microbiota homeostasis.

Gastrodin is therefore notable for its potential to intervene in RA-associated synovial pathology through immunometabolic and cell-death-related mechanisms, particularly H3K9 lactylation and ferroptosis. By contrast, the available evidence for UC is mainly derived from GEB-derived polysaccharides, whose protective effects involve inflammasome suppression and intestinal barrier preservation. Future studies should distinguish the contributions of individual constituents and determine whether these preclinical mechanisms can be translated into clinically effective interventions.

### 3.6 *Perilla frutescens* (L.) Britt.

*Perilla frutescens* (L.) Britt. is a widely consumed MEP whose leaves, stems, and seeds have long been used in dietary and traditional medicinal practices across East Asia, with a cultivation history spanning more than 2,000 years (99). Phytochemical studies have shown that *Perilla frutescens* contains diverse bioactive constituents, including flavonoids, phenolic acids, terpenoids, and essential oils (100).

In RA-related studies, methoxyflavanone isolated from *Perilla frutescens* suppressed CIA progression by negatively regulating T-cell receptor signaling and promoting Akt activation, thereby inhibiting pathogenic Th17 responses (101). Given the pivotal role of Th17/Treg imbalance in RA pathogenesis, these findings suggest that Perilla-derived flavonoids alleviate autoimmune arthritis by modulating Th17-associated adaptive immune responses rather than merely exerting nonspecific anti-inflammatory effects. In a psoriasis mouse model, essential oil extracted from *Perilla frutescens* alleviated psoriasis-like skin lesions, reduced the levels of IL-6, IL-1, IL-23, and IL-17, and suppressed NF-κB-related inflammatory activity, thereby attenuating inflammatory responses (102). This finding suggests that Perilla may interfere with the IL-23/IL-17 axis, a key pathogenic pathway in psoriasis. Interestingly, a recent study further demonstrated that extracellular vesicle-like particles derived from Perilla leaves carrying pab-miR-396a-5p alleviated psoriasis through modulation of IL-17 signaling (103). This observation extends the mechanistic profile of Perilla beyond conventional phytochemicals to plant-derived vesicle-mediated cross-kingdom immune regulation.

The immunoregulatory profile of *Perilla frutescens* is therefore closely linked to Th17/IL-17-associated inflammation, with evidence supporting its relevance to RA and psoriasis. The identification of Perilla-derived extracellular vesicle-like particles also provides a novel mechanistic direction for future investigation. Nevertheless, the current evidence is predominantly preclinical, and further studies are required to clarify the active constituents, delivery mechanisms, safety, and clinical applicability of Perilla-based interventions.

### 3.7 *Sesamum indicum* L.

*Sesamum indicum* L. belongs to the Pedaliaceae family and is one of the major traditional oil crops cultivated in China (104). Sesame seeds and sesame oil contain diverse bioactive components, including lignans, unsaturated fatty acids, and phenolic compounds, many of which exhibit anti-inflammatory and antioxidant activities (105). In an adjuvant-induced arthritis rat model, sesame oil reduced paw inflammation and oxidative stress, decreased RA-associated markers and inflammatory eicosanoids, and lowered the levels of IL-1β, IL-6, monocyte chemoattractant protein-1 (MCP-1), and TNF-α (106). Importantly, native sesame oil, but not oil depleted of minor components, reduced arthritis severity and bone loss, suggesting that its minor bioactive constituents contribute substantially to its anti-arthritic activity (106).

Evidence in MS provides both clinical and experimental support for the immunomodulatory effects of sesame oil. In a randomized clinical study, adjunctive sesame oil administration in patients receiving interferon beta-1a increased IL-10 levels and reduced IFN-γ, nitric oxide (NO), TNF-α, and lymphocyte proliferation compared with interferon beta-1a treatment alone (107). In an EAE mouse model, daily oral administration of sesame oil reduced clinical symptoms and delayed disease onset, accompanied by decreased IFN-γ production and increased IL-10 production in splenic mononuclear cells (108).

Evidence for sesame oil remains more limited than that for several other MEP-derived interventions discussed above; however, its antioxidant activity and modulation of Th1-associated inflammatory responses provide a rationale for further investigation in RA and MS. Additional clinical studies are required to identify the responsible bioactive constituents, establish effective formulations and doses, and confirm its therapeutic efficacy in ADs.

### 3.8 Glycyrrhiza uralensis

*Glycyrrhiza uralensis* belongs to the Fabaceae family, and its roots are rich in triterpenoids and flavonoids, which exhibit antioxidant and anti-inflammatory properties (109). Available studies have linked licorice-derived interventions to high mobility group box 1 (HMGB1) mediated inflammation in lupus nephritis, synovial inflammation and angiogenesis in RA, and Th1-associated neuroinflammation in EAE (110–113).

In a model of lupus nephritis, extracellular HMGB1 facilitates macrophage activation by promoting the accumulation of activated lymphocyte-derived DNA in endosomes. Glycyrrhizin alleviated nephritis in activated lymphocyte-derived DNA (ALD-DNA)-induced lupus mice by blocking extracellular HMGB1, as reflected by reductions in inflammatory cytokines, anti-double-stranded DNA antibodies, urine protein, and glomerular IgG and C3 deposition (111). Previous studies have further demonstrated that HMGB1 plays a central role in sterile inflammation and immune-mediated tissue injury, supporting its relevance in chronic autoimmune pathology (110). These findings identify HMGB1 inhibition as a disease-relevant mechanism by which licorice-derived compounds may protect against lupus-associated renal injury.

In RA models, liquiritin isolated from *Glycyrrhiza uralensis* alleviated arthritic manifestations through suppression of inflammation, MAPK signaling, and angiogenesis (112). *In vitro*, liquiritin inhibited IL-1β-induced RA-FLS proliferation, promoted apoptosis, reduced the Bcl-2/Bax ratio, decreased vascular endothelial growth factor (VEGF) expression, and suppressed c-Jun N-terminal kinase (JNK) and p38 phosphorylation; *in vivo*, it also induced apoptosis in synovial tissues (112). Thus, the anti-arthritic activity of liquiritin may involve not only attenuation of inflammatory signaling but also restriction of pathological synovial expansion and angiogenesis. In EAE models, ethanol extract of Glycyrrhizae Radix reduced IFN-γ^+^ Th1/Tc1 cell populations, cell adhesion molecule expression, and the secretion of IFN-γ and IFN-γ-induced protein 10 (IP-10). In IFN-γ-stimulated BV2 microglial cells, the extract also reduced NO, TNF-α, and IP-10 production, while in EAE mice it suppressed antigen-specific Th1/Tc1 responses (113). These findings suggest that licorice root extract may attenuate autoimmune neuroinflammation by suppressing Th1/Tc1-associated immune responses and microglial inflammatory activity, processes that are relevant to inflammatory tissue injury in MS (32, 114).

Licorice-derived interventions exhibit disease-specific mechanisms of action. Glycyrrhizin alleviates HMGB1-associated renal inflammation in lupus nephritis, whereas liquiritin suppresses synovial inflammation and angiogenesis in RA. In EAE, Glycyrrhizae Radix extract attenuates Th1/Tc1-associated neuroinflammatory responses. However, current evidence is largely based on experimental studies, and further investigation is needed to clarify the contributions of individual constituents and establish standardized formulations, safety profiles, and clinical efficacy.

### 3.9 Ganoderma lucidum

*Ganoderma Lucidum* (*G. Lucidum*) is a medicinal mushroom widely used in East Asian countries, including China and Japan, for immune regulation (115). The bioactive constituents of *G. Lucidum* exceed 400 compounds and include triterpenoids, polysaccharides, and nucleosides, which exert anti-inflammatory effects through inhibition of inflammatory mediator release and modulation of cellular signaling pathways (116).

In a CIA rat model, *G. Lucidum* polysaccharide peptide effectively alleviated RA symptoms in CIA rats by inhibiting the NF-κB and MAPK pathways (117). Additionally, *G. Lucidum* spore oil significantly reduced clinical arthritis scores, alleviated joint inflammation and cartilage damage, and inhibited IL-6 expression in CIA mice (118). Beyond general suppression of inflammation, *G. Lucidum* spore powder was shown to alleviate RA-associated pain hypersensitivity by inhibiting neutrophil accumulation, suppressing N1 neutrophil polarization, and reducing ROS generation (119). These findings suggest that neutrophil-driven oxidative injury may represent an important mechanism underlying the effects of *G. lucidum* in autoimmune arthritis. Evidence regarding MS-related neurological injury remains limited. In a cuprizone-induced demyelination mouse model, the fruiting bodies of medicinal mushrooms including *G. lucidum* inhibited demyelination and improved motor dysfunction (120). Because this model primarily reflects toxin-induced demyelination rather than autoimmune neuroinflammation, these results provide preliminary evidence of potential neuroprotective activity but do not directly establish immunomodulatory efficacy in MS.

By comparison, the intestinal protective effects of *G. lucidum* in colitis are supported by more clearly defined mechanisms. In UC models, β-D-glucans from *G. Lucidum* demonstrated protective activity by alleviating characteristic UC symptoms through regulation of inflammatory cytokine levels, modulation of intestinal immune responses, improvement of gut microbiota composition and short-chain fatty acid metabolism, and alteration of gene expression in colonic epithelial cells (121). In addition, ganoderic acid A, a major triterpenoid component of *G. lucidum*, protected against colitis by maintaining epithelial and mucus layer integrity, regulating tryptophan metabolism, enhancing aryl hydrocarbon receptor (AhR) activity, and modulating microbiota-associated metabolites (122, 123). These findings indicate that modulation of the gut microbiota-tryptophan metabolite-AhR axis, together with preservation of mucosal barrier integrity, may constitute a central intestinal protective mechanism of *G. lucidum*-derived constituents.

*G. lucidum*-derived interventions therefore show distinct mechanistic priorities across disease contexts. In RA, their effects involve suppression of inflammatory signaling and regulation of neutrophil-mediated oxidative injury, whereas in colitis, their protective actions are more closely associated with barrier preservation and modulation of the microbiota-metabolite-AhR axis. Future studies should distinguish the contributions of individual bioactive constituents and evaluate their disease-specific efficacy and safety in clinical settings.

### 3.10 Radix Astragali

*Radix Astragali* is one of the most widely used traditional medicinal and edible materials in China and exhibits diverse pharmacological activities, including immunomodulatory, antioxidant, and anti-inflammatory effects (124, 125). Its major bioactive constituents, such as astragalosides, flavonoids, and polysaccharides, have been reported to regulate immune responses and metabolic homeostasis through multiple signaling pathways (125).

In experimental AM rat models, *Radix Astragali* improved myocardial contractile function, reduced myocardial inflammation and fibrosis, and exerted cardioprotective effects by suppressing lymphocyte proliferation and restoring the balance between Th1 and Th2 cytokines (126). These findings suggest that correction of Th1/Th2-associated adaptive immune imbalance may contribute to the protective effects of *Radix Astragali* against autoimmune myocardial injury. In RA-related studies, a metabolomics-based investigation using urine and serum analyses demonstrated that *Radix Astragali* alleviated arthritis severity in AIA rats through regulation of tryptophan and phenylalanine metabolism, accompanied by reduced IL-1β, TNF-α, and MDA levels together with increased SOD activity (127). These findings indicate that *Radix Astragali* may attenuate RA-associated inflammation by modulating amino acid metabolism and restoring redox homeostasis.

The available evidence therefore places *Radix Astragali* as a potential regulator of immunometabolic disturbances in ADs. Its effects in experimental myocarditis are mainly associated with restoration of Th1/Th2 balance, whereas its activity in arthritis models appears to involve amino acid metabolism and oxidative stress regulation. However, current evidence remains predominantly preclinical, and further studies are needed to confirm its active constituents, pharmacological targets, and clinical applicability.

### 3.11 Comparative perspective and added value of this review

The representative MEPs discussed above show both overlapping and disease-specific immunomodulatory patterns. Previous reviews have provided valuable summaries of natural products, medicinal plants, dietary phytochemicals, or selected plant-derived compounds in autoimmune and inflammatory diseases. However, many of these reviews have focused on broad natural-product categories, single phytochemical classes, individual diseases, or isolated signaling pathways. By contrast, the present review focuses specifically on MEPs, a group of interventions with both dietary and medicinal relevance, and organizes them according to their major immune targets, disease contexts, and evidence boundaries.

This comparative perspective helps clarify that MEP-derived interventions should not be viewed simply as nonspecific anti-inflammatory agents. Some mechanisms are shared across different diseases, including NF-κB, MAPK, JAK/STAT, NLRP3 inflammasome signaling, Th17/Treg imbalance, oxidative stress, and barrier dysfunction. Other mechanisms appear more disease- or tissue-context dependent, such as HMGB1-associated renal inflammation in lupus nephritis, histone lactylation and ferroptosis in RA-associated synovial pathology, neutrophil-mediated oxidative injury in inflammatory arthritis, Th1/Th2-associated myocardial inflammation in AM, and microbiota–metabolite–AhR-associated intestinal protection in colitis. These distinctions are summarized in **Table 1** and **Figure 1**, which provide a cross-disease framework for comparing the principal mechanisms of representative MEP-derived interventions.

The added value of this review therefore lies in integrating plant-specific evidence with disease-specific immune pathology. Instead of ranking MEPs by presumed efficacy, which is currently difficult because of differences in models, preparations, doses, and outcome measures, this review emphasizes how different MEP-derived interventions may act through distinct but partially overlapping immune-regulatory networks. This framework may help readers identify which MEPs are more closely associated with particular immune targets or disease contexts, while also recognizing that most mechanistic evidence remains preclinical and requires further validation.

## 4 Conclusion

In conclusion, MEPs provide a rich source of bioactive compounds with potential to modulate immune dysfunction in ADs. The studies reviewed here show that their effects are mainly associated with suppression of excessive inflammatory signaling, regulation of immune-cell imbalance, control of oxidative stress, and protection of tissue barriers. Rather than acting through a single pathway, many MEPs appear to regulate partially overlapping immune networks that vary across disease contexts. At the same time, some MEPs act through more specific mechanisms, such as inhibition of HMGB1-associated inflammation, regulation of histone lactylation and ferroptosis in synovial pathology, modulation of neutrophil activation, and restoration of intestinal microbiota and barrier homeostasis. These findings suggest that MEPs may be particularly useful for addressing the complex and heterogeneous immune disturbances involved in ADs.

However, the strength of evidence varies substantially among different MEPs and disease settings. Much of the current literature is based on cellular experiments and animal models, whereas clinical evidence remains limited and uneven. In many cases, therapeutic implications are inferred from changes in inflammatory mediators or disease scores in preclinical models rather than confirmed patient benefit. In addition, few studies have directly compared different MEP-derived compounds or examined whether their reported mechanisms are reproducible across disease models. Therefore, the available data support their therapeutic potential, but are not yet sufficient to establish most MEPs as clinically validated interventions for ADs.

Several practical issues must also be resolved before MEP-derived interventions can be translated into routine clinical use. Many active compounds have poor oral bioavailability, rapid metabolism, or unstable pharmacokinetic properties. Variability in plant sources, extraction procedures, chemical composition, dosage, and formulation further complicates comparison between studies and limits reproducibility. Safety evaluation, interactions with existing immunomodulatory treatments, quality-control standards, and regulatory requirements also remain insufficiently addressed. Future studies should therefore move beyond descriptive pharmacological observations and focus on standardized preparations, clearly defined active constituents, mechanism-guided comparisons, pharmacokinetic and safety assessment, and well-designed clinical trials. At present, MEPs should be regarded as promising sources of adjunctive immunomodulatory strategies rather than replacements for established therapies. A more rigorous and clinically oriented research framework will be essential to translate their mechanistic promise into reliable therapeutic benefit for patients with ADs.
